# Risk factors for avascular necrosis of the femoral head after developmental hip dislocation reduction surgery and construction of Nomogram prediction model

**DOI:** 10.1186/s12891-024-07575-y

**Published:** 2024-06-14

**Authors:** Zidan Tang, Rong Li, Chan Lu, Na Ma, Rui Xie, Xiaopeng Kang, Xinhao Chen, Han Yang, Yong Hang, Jun Li, You Zhou

**Affiliations:** 1 Graduate School, Kunming Medical University, Kunming, 650500 China; 2 Department of Obstetric Ultrasound, The First Affiliated Hospital of Kunming Medical University, Kunming, 650032 China; 3 Department of Orthopedics, Kunming Children’s Hospital, No. 288, Qianxing Road, Xishan District, Kunming, Yunnan, 650100 China

**Keywords:** Developmental dysplasia of the hip, Casting, Avascular necrosis, Risk factors

## Abstract

**Background:**

To analyze the risk factors for the development of avascular necrosis (AVN) of the femoral head after reduction surgery in children with developmental hip dysplasia (DDH), and to establish a prediction nomogram.

**Methods:**

The clinical data of 134 children with DDH (169 hips) treated with closure reduction or open reduction from December 2016 to December 2019 were retrospectively analyzed. Independent risk factors for AVN after DDH reduction being combined with cast external immobilization were determined by univariate analysis and multivariate logistic regression and used to generate nomograms predicting the occurrence of AVN.

**Results:**

A total of 169 hip joints in 134 children met the inclusion criteria, with a mean age at surgery of 10.7 ± 4.56 months (range: 4–22 months) and a mean follow-up duration of 38.32 ± 27.00 months (range: 12–94 months). AVN developed in 42 hip joints (24.9%); univariate analysis showed that the International Hip Dysplasia Institute (IHDI) grade, preoperative development of the femoral head ossification nucleus, cartilage acetabular index, femoral head to acetabular Y-shaped cartilage distance, residual acetabular dysplasia, acetabular abduction angle exceeding 60°, and the final follow-up acetabular index (AI) were associated with the development of AVN (*P* < 0.05). Multivariate logistic regression analysis showed that the preoperative IHDI grade, development of the femoral head ossification nucleus, acetabular abduction angle exceeding 60°, and the final follow-up AI were independent risk factors for AVN development (*P* < 0.05). Internal validation of the Nomogram prediction model showed a consistency index of 0.833.

**Conclusion:**

Preoperative IHDI grade, preoperative development of the femoral head ossification nucleus, final AI, and acetabular abduction angle exceeding 60° are risk factors for AVN development. This study successfully constructed a Nomogram prediction model for AVN after casting surgery for DDH that can predict the occurrence of AVN after casting surgery for DDH.

## Background

Developmental Dysplasia of the Hip (DDH) is a developmental alteration in pediatric orthopedics, with an estimated incidence ranging from 1.5 to 20 per 1,000 [1, 2]. The primary objective of DDH treatment is to achieve and maintain concentric reduction of the femoral head within the acetabulum. For children under 24 months of age, closed reduction (CR) or surgical open reduction (OR) surgery (with casting) is the preferred method, with a reported success rate of up to 90% [3]. However, these surgical methods carry various complications, with avascular necrosis (AVN) being the most severe and prevalent, affecting up to 47% of patients [2, 4]. AVN can significantly impact hip function and increase the likelihood of secondary surgeries.

The exact etiology of AVN remains elusive, and previous studies have identified factors that may correlate with AVN development following DDH reduction surgery, including a high abduction angle of the hip, open reduction, absence of ossification nucleus preoperatively, as well as protective factors such as early age at surgical reduction and presence of ossification nucleus [5–10]. Nevertheless, the individual contribution of each factor to AVN remains unclear, and there is currently no effective prediction model available.

The Nomogram model is a visual representation of various risk factors that enables more accurate individual predictions [11]. This model has been widely utilized in prognostic evaluations for various diseases, yet to our knowledge, no study has utilized the Nomogram model to predict AVN occurrence following DDH reduction surgery. The objective of this study is to identify independent risk factors associated with AVN development following DDH reduction surgery and establish a Nomogram prediction model. This model aims to enable early identification and intervention for DDH patients at a heightened risk of developing AVN.

## Methods

### Clinical data

This retrospective study was performed in accordance with the guidelines of the Ethical Committee on Human and Animal Experiments of Kunming Children’s Hospital (2024-05-005-K01). Informed consent was obtained from parents of participants as children are involved in the study. All experimental protocols were approved by Kunming Children’s Hospital licensing committee. Patients with DDH who underwent closed reduction or OR surgery at our hospital between December 2016 and December 2019 were enrolled in this study.

Inclusion criteria: (1) All patients were children aged ≤ 24 months; (2) Diagnosis of DDH was made based on medical history, physical examination, and X-ray imaging; (3) All patients underwent primary CR or OR surgery with casting; (4) Follow-up duration was ≥ 1 year; (5) Complete imaging data and postoperative hip magnetic resonance imaging (MRI) were available.

Exclusion criteria: (1) Patients with neuromuscular diseases or traumatic hip dislocation; (2) Patients who experienced failure of primary CR or OR surgery with casting; (3) Patients with incomplete follow-up data.

### Surgical methods

**Fig. 1:**
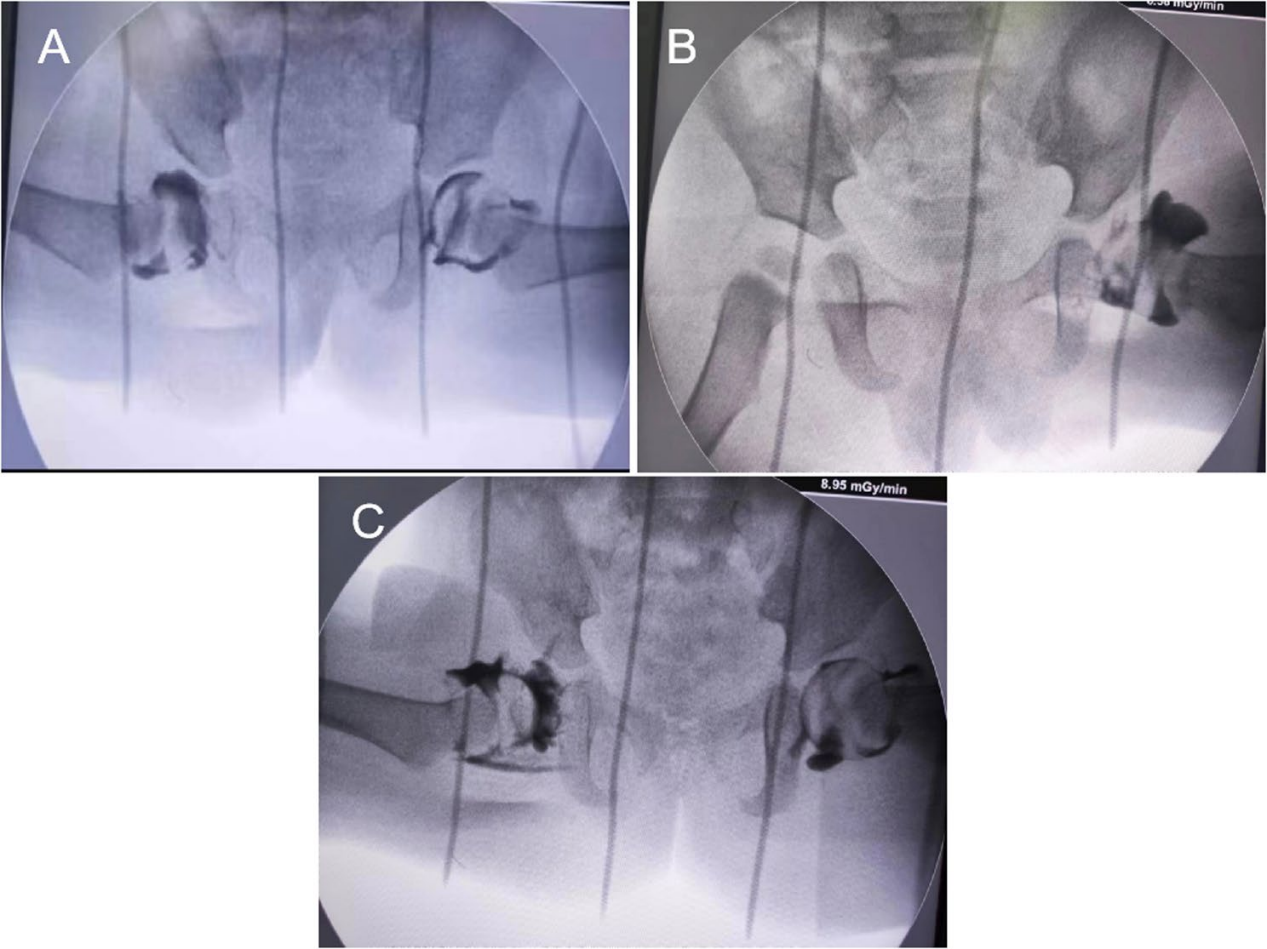
The intraoperative hip hip arthrogram injection of the patient. **A**-**B** Good femoral head coverage; **C** Poor femoral head coverage

Based on the X-ray at the last follow-up, we classified the patients into two groups: AVN group (group A) and non-AVN group (group B) according to Kalamchi-MacEwen classification [12]. The preoperative dislocation degree of each patient was graded using the IHDI (Table 1). Under general anesthesia, all patients underwent hip reduction using Ortolani manipulation, and hip arthrogram injection was conducted intraoperatively. The reduction achieved was evaluated as “stable” and “safe” based on the coverage of the femoral head observed during angiography. According to Bowen’s arthrogram classification, if angiography showed good coverage of the femoral head (Fig. 1A and B), CR was performed; otherwise, if the coverage of the femoral head was poor (Fig. 1C), OR was conducted. If hip joint instability was observed, OR was immediately performed.

**Table 1:** IHDI classification evaluation criteria

Type	Evaluation criteria
I	H point is on or inside the P line
II	H point is outside the P line, on or inside the D line
III	H point is outside the D line, on or below the H line
IV	H point is above the H line

All patients underwent OR through a medial approach. During the operation, the joint capsule was incised in a “T” shape, the transverse ligament was transected, and the enlarged round ligament was resected. The hypertrophic adipose tissue in the acetabulum was also removed. After reduction, hip-knee-ankle splint fixation in human position was applied for 3 months, followed by external fixation support for 3–6 months.

### Evaluation indicators

The age, gender, surgical method, and history of previous bracing treatment of all patients were recorded. Based on the preoperative X-ray, the preoperative dislocation degree of acetabulum (classified by IHDI) and preoperative acetabular index (AI) were recorded for each patient. It was also recorded whether there was ossification of the femoral head before surgery. The MRI scan within 48 h after surgery was recorded, and the abduction angle of the hip joint, cartilage acetabular index (CAI), distance from femoral head to acetabular cartilage (FTD), and evaluation of acetabular labrum were measured; the acetabular index and central edge angle at the last follow-up were also recorded. All patients’ radiographic data were observed and measured by two senior radiologists.

### Statistics

Statistical analysis was performed using SPSS 25.0 software (SPSS corp., Chicago, IL, USA). Normal distributed data were expressed as mean ± standard deviation, while non-normal distributed data were expressed as median. Categorical data were compared using chi-square test or Fisher’s exact test. For normally distributed continuous variables, the Student’s t-test was utilized, and for non-normally distributed continuous variables, the rank sum test was used. A multivariate logistic regression analysis was conducted to determine the factors associated with AVN occurrence. Risk factor variable assignments for AVN are shown in Table 2. The threshold for statistical significance was set at *P* < 0.05.

**Table 2:** AVN’s influence factor variable assignment

Variable	Assignment
IHDI classification	IHDI II = 2, IHDI III = 3, IHDI IV = 4
Ossification nucleus of the femoral head	Yes = 1, No = 2
Hip abduction angle	< 60 = 1, ≥ 60 = 2
AVN	Occurred = 1, Not occurred = 2
Prior treatment history	Yes = 1, No = 2
Affected side	Left = 1, Right = 2, Both = 3
Surgical approach	Closed reduction = 1, Open reduction = 2
Gender	Female = 1, Male = 2

The nomogram was created using the multivariable model in R 3.6.0. ROC curves were generated, and the AUC with 95% CI was calculated to assess the model’s discrimination. The nomogram was also internally validated using tenfold cross-validation. Calibration plots were created, comparing the predicted and actual observed probabilities. Finally, DCA was used to evaluate the net benefit of the model.

## Results

### General information

A total of 134 patients (169 hips) with DDH met the inclusion criteria, including 11 males (8.46%) and 123 females (91.54%); 57 hips (33.73%) on the left, 41 hips (24.27%) on the right, and 36 hips (21.36%) in both sides; 43 hips (25.40%) received Pavlik harness before hip reduction surgery; 102 hips (60.36%) underwent CR, and 67 hips (40.24%) underwent OR; preoperative IHDI classification: 51 hips in type II, 75 hips in type III, and 43 hips in type IV. The mean preoperative AI was 37.47 ± 6.28, with a mean final AI of 24.27 ± 0.53; preoperative ossification nucleus was present in 114 hips (67.04%), and no ossification nucleus was present in 55 hips (32.96%); postoperative residual dysplasia was observed in 45 hips (26.69%); the average age at surgery was 10.7 ± 4.56 months (range 4–22 months) (Table 3).

**Table 3:** Basic clinical informations of the patients

Variable	AVN (42 hips)	Non-AVN (127 hips)
Surgical age	11.42 ± 4.50	10.50 ± 4.60
**Gender**		
Male	6 (14.3%)	12 (9.4%)
Female	36 (85.7%)	115 (90.6%)
**Prior harness treatment**		
Yes	11 (26.2%)	32 (25.2%)
No	31 (73.8%)	95 (74.8%)
**IHDI classification**		
Type II	5 (11.9%)	46 (36.2%)
Type III	19 (45.2%)	56 (44.1%)
Type IV	18 (44.9%)	25 (19.7%)
**Preoperative ossification nucleus**		
Present	21 (50%)	93 (73.2%)
Absent	21 (50%)	34 (26.8%)
**Side affected**		
Left	14 (33.3%)	42 (33.1%)
Right	9 (21.4%)	34 (26.8%)
Bilateral	19 (45.3%)	51 (40.1%)
**Surgical approach**		
Closed	24 (57.1%)	78 (61.4%)
Open	18 (22.9%)	49 (38.6%)
**Labral inversion**		
Yes	22 (52.4%)	45 (35.4%)
No	20 (47.6%)	82 (64.6%)
Preoperative AI angle	37.46 ± 6.28	36.59 ± 5.71
Abduction angle	59.90 ± 8.13	58.13 ± 4.32
residual hip dysplasia	25 (59.5%)	20 (15.7%)

### Risk factors for AVN: clinical characteristics

According to the K&M classification, postoperative femoral head necrosis occurred in 42 hips, representing a necrosis rate (NR) of approximately 24.9%. The NR in male patients was 33.3% (6/18), and NR in the female patients was 23.8% (36/151). The NR in unilateral hip group was 23.3% (23/99), with bilateral group of 27.1% (19/70). In terms of treatment methods, the CR group’s NR was 23.5% (24/102), and the OR’s NR was 26.9% (18/67) (Table 4).

**Table 4:** Risk factors for AVN: clinical features

Variable	Total	AVN group	Non-AVN group	p-value
Surgical approach	169			0.649
Closed	102	24	78	
Open	67	18	49	
Prior harness treatment history				0.919
Yes	43	11	32	
No	126	31	95	
Gender				0.388
Male (hips)	18	6	12	
Female (hips)	151	36	115	
Side affected (hips)				0.772
Left	56	14	42	
Right	43	9	34	

There were no significant differences in gender, age, or affected side between the AVN group and the non-AVN group. Additionally, there were no significant differences in the incidence of AVN based on surgical approach or prior Pavlik harness treatment history between the two groups.

### Risk factors for AVN: imaging characteristics

The average FTD of the hip joint was 2.34 ± 0.15 mm (range 0–8.1 mm). The FTD was wider in the AVN group (3.15 ± 1.56 mm) compared to the non-AVN group (2.07 ± 1.91 mm) (*p* = 0.001). Among patients with acetabular labrum inversion, 22 developed AVN and 45 did not develop AVN (*p* = 0.06). The average abduction angle for all patients was 58.86 ± 4.27 mm, with an average abduction angle of 59.90 ± 8.13° in the AVN group and an average abduction angle of 58.13 ± 4.32° in the non-AVN group (*p* = 0.290). The average cartilage acetabular index for all patients was 13.02 ± 4.76°, with an average cartilage acetabular index of 15.43 ± 5.75°in the AVN group and an average cartilage acetabular index of 12.22 ± 6.12° in the non-AVN group (*p* = 0.003) (Table 5).

**Table 5:** Risk factors for AVN: imaging features

Variable	Total	AVN group	Non-AVN group	P-value
FTD	2.34 ± 0.15	3.15 ± 1.56	2.07 ± 1.91	0.001
Labral inversion	67	22	45	0.061
External rotation angle	58.86 ± 4.27	59.90 ± 8.13	58.13 ± 4.32	0.29
Cartilage hip acetabular index	13.02 ± 4.76	15.43 ± 5.75	12.22 ± 6.12	0.003
IHDI classification				0.002
Type II	51	5	46	
Type III	75	19	56	
Type IV	43	18	25	
Preoperative ossification nucleus				0.006
Present	114	21	93	
Absent	55	21	34	
Preoperative average AI angle	37.47 ± 6.28	37.46 ± 6.28	36.59 ± 5.71	0.404
Hip abduction angle ≥ 60°	76	25	51	0.008
Latest AI angle	24.27 ± 0.53	29.65 ± 2.57	22.79 ± 0.50	<0.01

In terms of the IHDI classification, the AVN incidence rate for type II was 9.8% (5/51), type III was 25.3% (19/75), and type IV was 38.2% (18/43) (*p* = 0.03). As the IHDI grade increased, the AVN incidence also increased, with the presence of ossification nucleus serving as a protective factor for AVN occurrence. The presence rate of ossification nucleus is 67% (114/169), with 50% in the AVN group and 73.2% in the Non-AVN Group (*p* = 0.006).

**Fig. 2:**
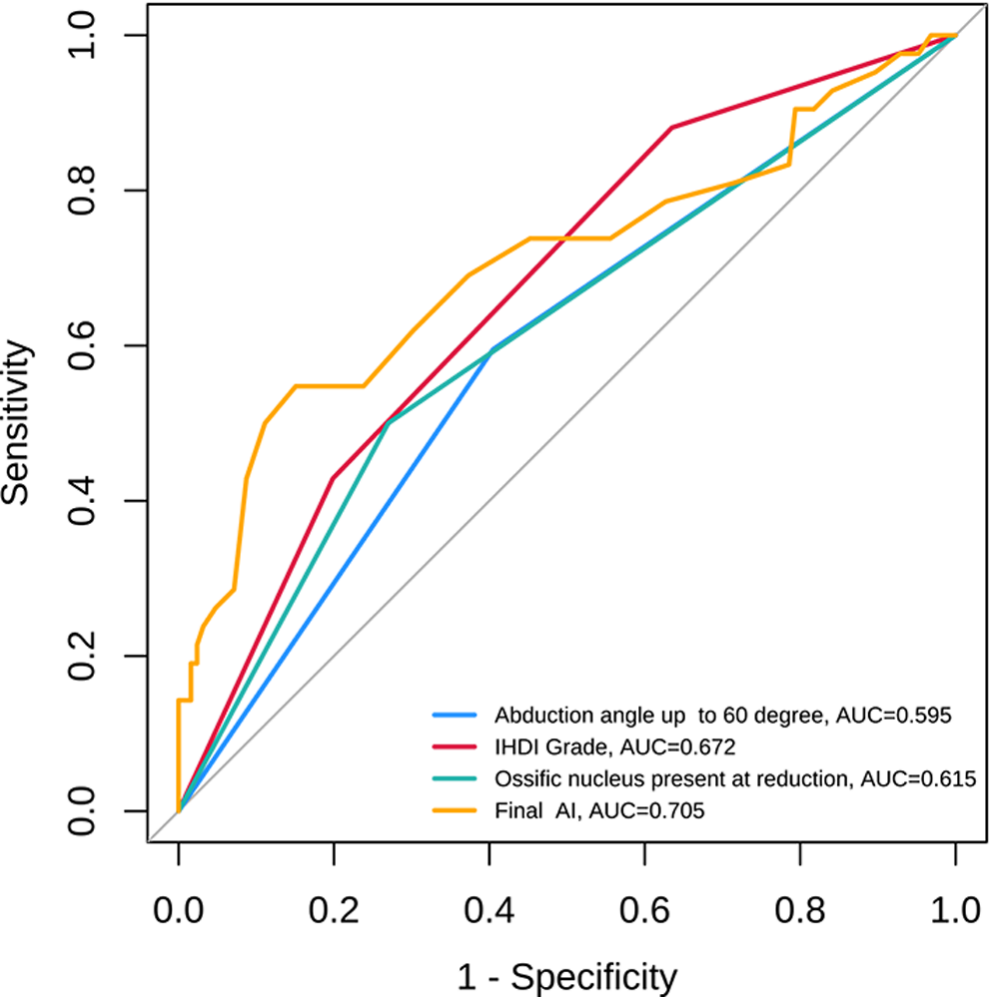
The ROC curve of hip abduction angle, IHDI classification, ossification nucleus and the last AI for predicting the occurrence of AVN after the treatment of developmental hip dislocation

Univariate analysis showed that IHDI classification, preoperative ossification nucleus development, cartilage acetabular index, FTD, final AI, and hip abduction angle ≥ 60° were associated with AVN occurrence; multivariate logistic regression analysis revealed that FTD and CAI were not significantly associated with AVN occurrence (*P* > 0.05), while preoperative ossification nucleus development, IHDI classification, hip abduction angle ≥ 60°, and final AI were independent risk factors for AVN occurrence (*P* < 0.05). The area under the ROC curve for the final AI was 0.871 (Fig. 2), indicating an optimal cutoff value of 27.7° (sensitivity 46%, specificity 85%) (Table 6).

**Table 6:** Logistic multifactorial analysis of risk factors of AVN

Variable	OR	95%CI	P-value
FTD	0.801	(.637, 1.006)	0.056
IHDI	0.42	(.221, .799)	0.008
Ossification nucleus	0.207	(.081, .528)	0.001
CAI	0.973	(.907, 1.044)	0.447
Hip abduction angle ≥ 60°	0.34	(0.138, .836)	0.019
Latest AI angle	0.882	(0.822, .946)	0

### Nomogram prediction model

**Fig. 3:**
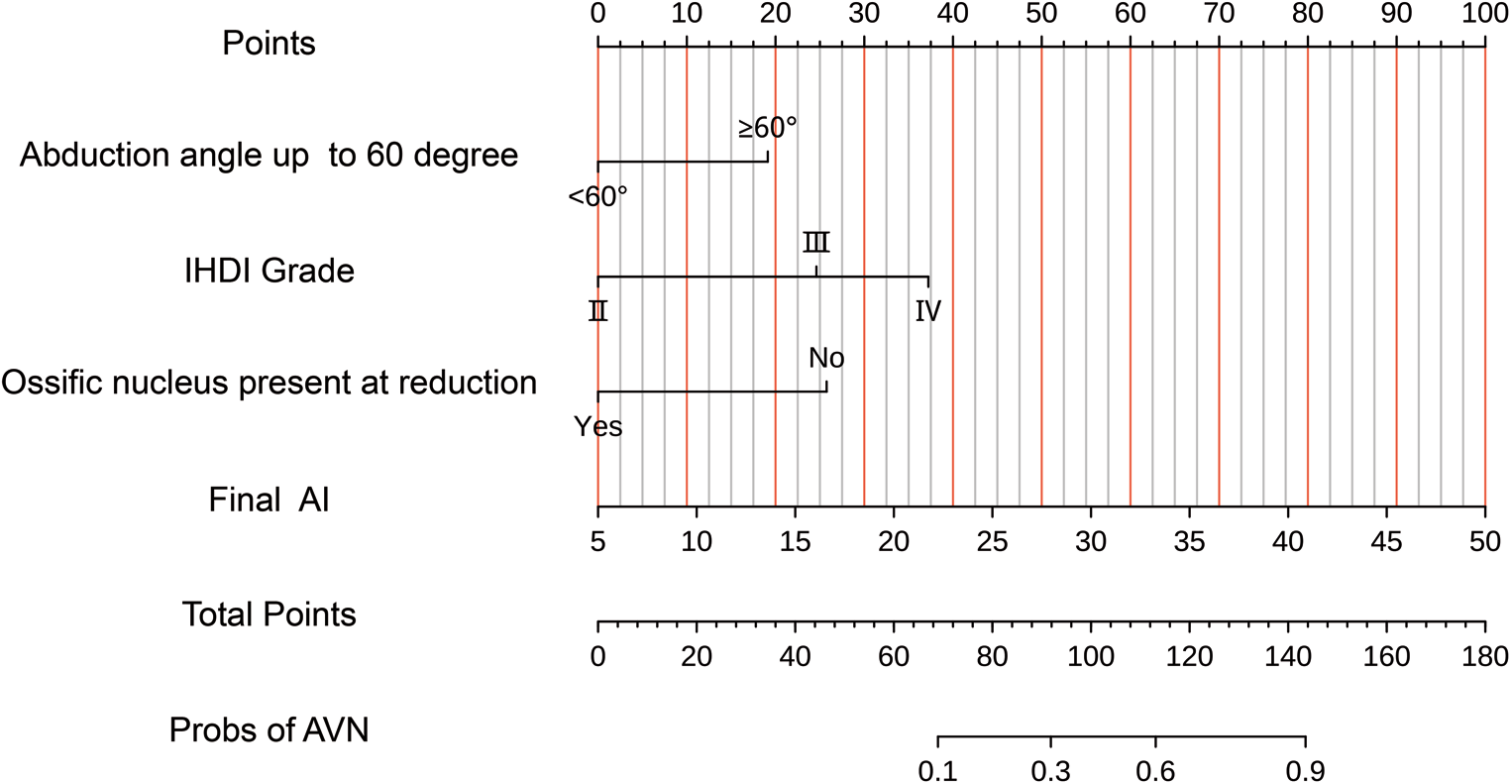
The Nomogram prediction model for the occurrence of AVN after the treatment of developmental hip dislocation

**Fig. 4:**
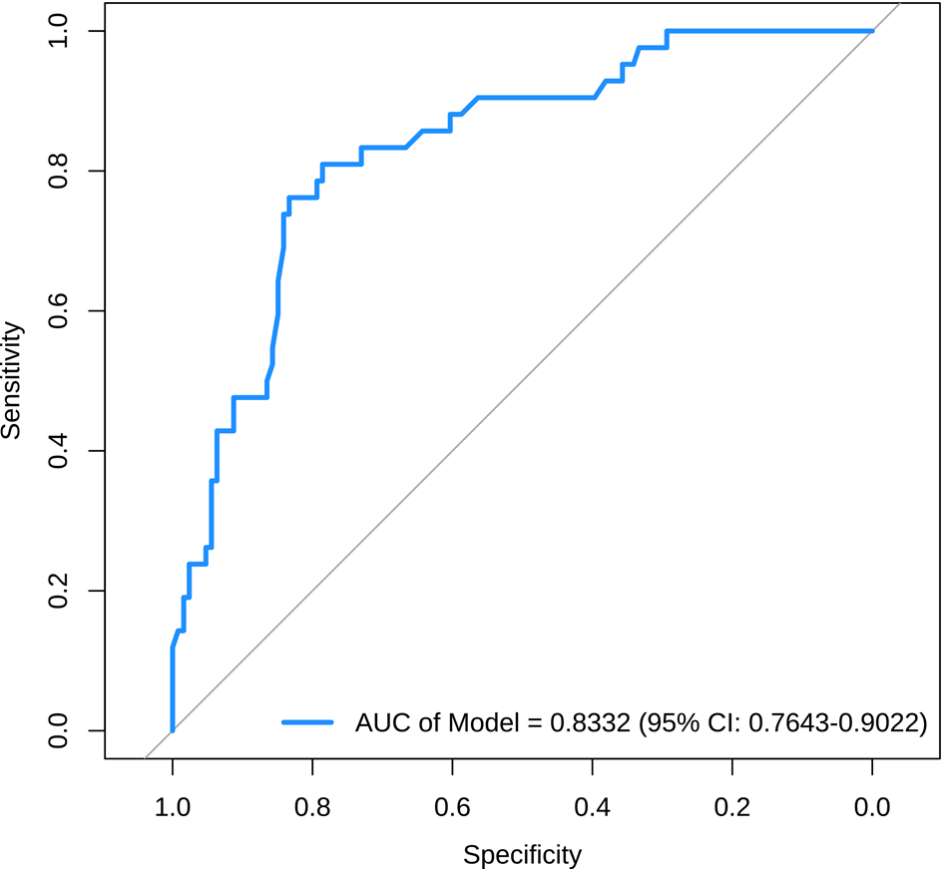
The ROC curve of the Nomogram prediction model

The Nomogram model was meticulously constructed by incorporating four independent risk factors: hip abduction angle, IHDI classification, ossification nucleus, and final AI. Figure 3 illustrates the internal data validation process, which yielded a calibration index (CI) of 0.833 (95% CI: 0.764, 0.902). This CI serves as a testament to the strong concordance between the Nomogram’s predictive capabilities and the actual observed outcomes (Fig. 4).

## Discussion

AVN is a significant complication that can occur following treatment for DDH [4, 13, 14]. Previous research has shown a wide variation in the incidence of AVN, which may be attributed to differences in the criteria used to define AVN, as well as debated factors that influence its occurrence [4, 13, 14]. Our current study aims to assess the factors that impact femoral head necrosis, including preoperative IHDI classification, development of the femoral head ossification nucleus, age at the time of surgery, gender, affected side, surgical method, previous treatment history, preoperative acetabular index, preoperative cartilage acetabular index, distance from the femoral head to the hip joint, and labral inversion. Our research indicates that the development of the femoral head ossification nucleus, IHDI classification, hip abduction angle ≥ 60°, and last acetabular index are independent risk factors for AVN.

In our study, we identified a statistically significant difference between preoperative IHDI classification and the occurrence of AVN, suggesting that preoperative IHDI classification is an important factor influencing the occurrence of AVN. The incidence of AVN in our research was 9.80% for IHDI grade II, 25.3% for IHDI grade III, and 38.2% for IHDI grade IV, aligning with the findings of Pang H, et al. [8, 15–18]. Higher degrees of preoperative dislocation may lead to more severe pathological changes in the hip, resulting in poor alignment between the femoral head and acetabulum. The sudden increase in pressure on the femoral head and acetabulum after reduction and changes in the internal mechanical environment of the hip joint postoperatively may contribute to the increased incidence of AVN. Conversely, although the pressure on the femoral head is reduced with higher degrees of dislocation, the increased tension on the blood vessels can easily cause ischemic necrosis, and the trauma experienced during reduction can easily damage the blood vessels [7, 12, 19].

Previous studies have highlighted the surgical approach and excessive abduction as major contributors to iatrogenic avascular necrosis (AVN). Several researchers have suggested that excessive hip abduction could compress the medial femoral circumflex artery or elevate hip joint pressure, potentially compromising the blood supply to the femoral head and leading to AVN [20, 21]. In our study, we found no statistically significant difference between abduction angle and AVN incidence. However, further investigation did reveal a statistically significant difference in AVN occurrence when hip abduction in children exceeded 60°. Considering that children aged 6 months to 4 years primarily rely on the medial femoral circumflex artery for blood supply to the femoral head, excessive abduction in an immobilized position may obstruct blood flow from this artery. Thus, we recommend keeping hip abduction below 60° when applying plaster fixes to children.

The negative correlation between age and AVN incidence in children under 6 months due to the development of femoral head microcirculation has been reported in previous literature. However, in our study, most children were over 6 months old at the time of surgery, and no statistical difference was found between AVN occurrence and age, suggesting that age does not act as a protective factor against AVN occurrence, consistent with Luhmann’s conclusions. It is important to note that magnetic resonance measurement of abduction angle is susceptible to bias, and when the hip flexion angle is less than 90°, it may lead to an increase in the measured abduction angle.

There is limited literature on the relationship between the femoral head-to-hip joint distance (FTD) and AVN. Different studies have reported varying acceptable distances between the femoral head and the acetabulum [15, 22]. In our study, we used magnetic resonance imaging to measure the FTD, and a multi-factor logistic regression analysis indicated that FTD had no significant effect on AVN occurrence (*p* > 0.05). Although the multi-factor analysis showed no statistical significance in AVN occurrence, the FTD was wider in the AVN group than in the non-AVN group in our study. Therefore, we suggest minimizing the distance between the femoral head and the acetabulum during casting.

In our study, simple open reduction was not an important factor in the development of AVN after casting. Previous studies have shown that open reduction can increase the risk of femoral head necrosis [14, 16, 17]. The literature reports that simple open reduction may cause damage to the medial femoral circumflex artery, and avascular necrosis of the femoral head may be the mechanism of AVN [14, 16, 17]. The medial femoral circumflex artery is located between the adductor and iliac muscles, passes through the anteromedial capsule of the hip joint, and is easily damaged when the joint capsule is incised along the medial approach. In recent reports, it is increasingly believed that the medial approach incision does not increase the occurrence of AVN. This is also supported by a 22-year follow-up study by Farsetti et al. [4]. Ergin and Novais et al. found that the medial approach incision did not increase the rate of AVN to 20% [23, 24]; Fisher et al. believes that the medial approach can cause damage to the medial femoral circumflex artery [25], but it does not cause femoral head necrosis. Their experiments found that damage to the medial femoral circumflex artery caused femoral head necrosis, which returned to normal after 4 months. There are also reports that open reduction can effectively reduce hip joint pressure by removing soft tissue and obstructing factors within and outside the joint capsule, and can be fixed with slight external rotation using a plaster cast, effectively reducing the pressure between the femoral head and acetabulum and reducing the occurrence of AVN [26]. There was no significant difference in the surgical method and the occurrence of AVN in our study.

In some studies, the presence of an ossification nucleus during reduction has a protective effect on the occurrence of AVN, suggesting delayed surgery to reduce the occurrence of AVN [27, 28]. They believe that the ossification nucleus may increase the mechanical strength of the femoral head and protect the blood supply of the epiphysis from exogenous compression. Although there are also many reports that the presence of a ossification nucleus during reduction does not reduce the occurrence of AVN. In our study, ossification had a protective effect on AVN. When there is no ossification nucleus in the femur, the blood supply of the cartilage epiphysis is a diffuse tubular network mainly distributed in the terminal arteries. Each blood vessel supplies a specific cartilage epiphysis region without anastomosis with other terminal arteries. At this time, femoral head ischemia and necrosis are prone to occur, leading to AVN. When ossification occurs, effective collateral circulation may be established at the epiphysis of the femoral head, making it better able to resist ischemia [7]. Some researchers believes that when ossification nucleus development is delayed, there may be obstacles in ossification nucleus development from the beginning [5, 9, 29]. The trabecular structure of the femoral head is fragile and prone to AVN. The development of ossification nuclei is also associated with dislocation. The higher the dislocation, the smaller the ossification nuclei and higher blood vessel tension are, increasing the probability of necrosis. Although there may be a protective effect of ossification nuclei on AVN, we do not support delaying reduction because delayed closed reduction may miss the optimal developmental time for acetabular development, leading to residual dysplasia. Residual dysplasia significantly reduces acetabular coverage of the femoral head, making it unable to receive effective stimulation and increasing the risk of AVN occurrence.

In this study, we found that the final AI was an independent risk factor for AVN after DDH reduction. The larger AI could lead to the higher probability of AVN occurrence. The larger AI refers to the smaller acetabular coverage of femoral head was, making it unable to receive effective stimulation, which may be an important reason for AVN occurrence. Similarly, development of femoral head and acetabulum is interrelated. The occurrence of AVN reduces stimulation of femoral head on acetabulum and affects acetabular development, leading to increased AI. Postoperative AI has rarely been reported in domestic and foreign literature as a factor influencing AVN occurrence. We need longer follow-up because DDH is in a stable state for AI at 2–4 years after reduction surgery.

Similar to other studies, AVN risk in this series was not affected by factors such as surgical age, gender, affected side or preoperative acetabular index. There is controversy about age as a risk factor for AVN occurrence. After our research analysis, AVN seems to have no impact on surgical age. Further research on gender and affected side found no significant relationship between gender or affected side and AVN occurrence. Although some reports have shown that male children have a higher risk of AVN, they cannot explain why this occurs [5, 9, 29].

In this study, we constructed a Nomogram prediction model using the independent influencing factors for the occurrence of AVN after DDH plaster immobilization as indicators and validated it using internal data, and the results showed a CI of 0.833 (95% CI (0.764, 0.902)), which suggests that this Nomogram prediction model is in good agreement with the actual observations. The limitations of this study lie in its single-center retrospective design, which may introduce selection bias, as well as the relatively small number of patient cases and the relatively short follow-up period. Moreover, type II AVN is not assessable on x-rays until typically at least age 6 and therefore this would be underreported with the follow up period supplied.

## Conclusions

In conclusion, this study provides valuable insights into the risk factors for AVN following DDH fixation and presents a predictive model for early detection. However, further research and long-term follow-up are essential to validate the findings and enhance the understanding of AVN occurrence in these patients.

## Data Availability

All the data used to support the findings of this study are included within the article.
